# The Lived Experience of Domestic Violence in Iranian HIV-Infected Women

**DOI:** 10.5539/gjhs.v7n5p43

**Published:** 2015-02-24

**Authors:** Nooredin Mohammadi, Hamid Emadi Kochak, Maryam Gharacheh

**Affiliations:** 1 Centre for Nursing Care Research, Department of Critical Care Nursing, Faculty of Nursing and Midwifery, Iran University of Medical Sciences, Tehran, Iran; 2 Iranian Research Centre for HIV/AIDS, Department of Infectious Disease, Faculty of Medicine, Tehran University of Medical Sciences, Tehran, Iran; 3 Department of Reproductive Health, Faculty of Nursing and Midwifery, Tehran University of Medical Sciences, Tehran, Iran

**Keywords:** lived experiences, HIV-infected women, domestic violence

## Abstract

Domestic violence is one of the most prevalent problems linked to HIV. Domestic violence in HIV-infected women has not been sufficiently explored, particularly in developing countries including Iran. This study aimed to explore the lived experience of domestic violence in Iranian HIV-infected women. A qualitative approach was used to conduct the study. Data were collected through semi-structured, in-depth interviews with ten HIV-infected women and were analyzed using content analysis. During the data analysis, four main themes emerged including, “regretful past”, “disappointing future”, “loneliness”, and “no other option”, which refer to the condition that the participants experienced in their lives due to challenges that mainly stem from the experience of HIV-related domestic violence. HIV infection can be a risk factor for domestic violence. Health care providers need to address domestic violence during the assessment of HIV-infected women and make appropriate referrals for abused women.

## 1. Introduction

HIV infection and domestic violence remain two major public health challenges influencing the lives of millions of women around the world (Aryal, Regmi, & Mudwari, 2012). Globally between fifteen and seventy one percent of women encounter domestic violence during their lifetime (Hatcher et al., 2013). In addition, women continue to be the fastest-growing population to be HIV-infected in many parts of the world (World Health Organization [WHO], 2013) and they currently account for fifty two percent of all HIV-infected people in developing countries (United Nations AIDS [UNAIDS], 2013).

Domestic violence is defined as a pattern of violent behaviors that occurs between two people in an intimate relationship such as marriage (Iliyasu, Abubakar, Babashani, & Galadanci, 2011). Being exposed to domestic violence is a complex phenomenon and a stressful life situation (Hayati, Eriksson, Hakimi, Högberg, & Emmelin, 2013) associated with experiences including, loss of self, being controlled, destruction, and struggle to recuperate from deep internal scars (Engnes, Liden, & Lundgren, 2012). For many women across the world, domestic violence is a daily occurrence (Hale & Vazquez, 2011).

Domestic violence has been documented as a culture-specific phenomenon and is influenced by religion and socio-cultural context (Pournaghash-Tehrani, 2011; Enwereji, 2007; Flinck, Paavilainen, & Astedt-Kurki, 2005). Iran shares the high prevalence of domestic violence with developed countries. Iranian society is still controlled by traditional values and patriarchal family structure (Abadi, Ghazinour, Nojomi, & Richter, 2012). According to Iranian civil law, men are considered as head of family and are responsible for supporting the family and women should obey their husbands (Sadeghi- Fassaei & Kamel-Ghalibaf, 2011). Although domestic violence is condemned and considered a crime, it is not easy to prosecute the perpetrators of violence. Thirty percent of married women experience at least one episode of severe physical violence during their marriage (Abadi et al., 2012). Consequently, many couples consider their conflicts to be a private issue, rendering them more unlikely to seek help and counseling, which can reinforce the cycle of domestic violence and ultimately result in negative health effects on the victims and the family as a whole (Pournaghash-Tehrani, 2011).

It is well established that domestic violence is related to HIV (Hatcher et al., 2013; Iliyasu et al., 2011; Li et al., 2014). Although the issue of domestic violence is increasingly focused on in the current literature, the issue of HIV-related domestic violence is not sufficiently discussed. HIV-infected women are almost three times as likely as uninfected women to experience domestic violence (UN Women, 2012). A study in Uganda revealed that twenty nine percent of HIV-infected women have experienced physical and sexual partner violence in the last 12 months (Osinde, Kaye, & Kakaire, 2011). Domestic violence may also hinder HIV-infected women from medical therapy and decrease their compliance and medication adherence (Schafer et al., 2012). Therefore, living with HIV is an important predictor of domestic violence against women and HIV-infected women are a vulnerable group for HIV-related domestic violence (Aryal et al., 2012) and the effect on them is more serious in terms of psychological, physical and pathological outcomes (Iliyasu et al., 2011).

Yet, HIV-related domestic violence has remained a hidden phenomenon (Hale & Vazquez, 2011) and there is limited knowledge to address the experience of domestic violence among women living with HIV. Given that the number of HIV-infected women is currently increasing and they appear to be at higher risk for domestic violence, it is necessary that lived experience of HIV-related domestic violence be focused on in research, policy and services to provide appropriate interventions and support to HIV-infected women. In addition, due to the socio-cultural nature of domestic violence, it is reasonable to suspect that the experience of HIV-related domestic violence in Iranian women might differ from women in other countries. Thus, this study used a qualitative approach to explore HIV-infected women´s experiences of domestic violence in Iran.

## 2. Method

A qualitative methodology was used to conduct the study. The aim of qualitative research is to illustrate and interpret real world phenomena in terms of the meanings subjects ascribe to them (Denzin & Lincoln, 2011). If there is little knowledge about the phenomenon or if this is fragmented, the inductive content analysis approach is recommended. Content analysis is a research technique for making valid inferences from texts to their contexts, with the objective of providing knowledge, new insights, and a representation of realities (Elo & Kyngas, 2008).

### 2.1 Participants

A purposive sampling technique was implemented to recruit ten participants from a counseling center for behavioral diseases, affiliated to Tehran University of Medical Sciences. Potential participants were recruited on the basis of the following criteria: HIV-infected women who were experiencing domestic violence and who agreed to participate in the study. Participants were married and spoke the Persian language. They were also living in the geographical border. Recruited participants were aged between 24 and 45 years. Seven participants had completed secondary education, and three did not complete secondary education. All participants were receiving medical care and length of time since HIV diagnosis was 1 to 10 years. The duration of their marriage ranged from 3 to 15 years. Three participant’s husbands were uninfected. The majority of participants had acquired HIV infection through their husbands. Three participants had become infected through unknown.

### 2.2 Data Collection

Data were collected through semi-structured, in-depth interviews from June 2013 to November 2013. The participants were asked to talk about their experiences of domestic violence. The starting questions were ‘Could you tell me about your relationship with your husband?; Do you often argue with your husband?; What feelings do you have when you experience violence?’. Probing questions also were used to clarify participants’ descriptions, such as, ‘When you said… what did you mean?; Could you explain more about that?’.

Ten participants were interviewed in a safe and private room in the counseling center for behavioral diseases by the principle researcher. Interview sessions lasted between 40 to 120 minutes. Although most of the participants had one interview session, four participants were interviewed twice. At the end of the interview session, participants received a gift card for their participation in the study. All interviews were audio-tape-recorded and transcribed verbatim in Persian language. Then each transcript was converted and saved to a separate Rich Text Format file and imported to MAXQDA 10 software.

### 2.3 Data Analysis

Data analysis was begun simultaneously with data collection. The data were analyzed using a conventional content analysis approach. To analyze data, the principle researcher read each transcript several times and looked for phrases from the participants that appeared particularly essential about the studied phenomenon; HIV-related domestic violence, and examined each sentence for meaning units. Then, transcripts were broken down into meaning units that were condensed, abstracted, and labeled with a code. Codes were categorized according to their semantic similarities. Sub-categories with similar incidents were then grouped together as categories and themes emerged. Finally, the themes were reviewed and revised by the research team members and consensus on the main themes was reached.

### 2.4 Ethical Consideration

Ethical approval for this study was obtained from the Ethics Committee of the Research Deputy in Tehran University of Medical Sciences. A written informed consent was obtained from participants prior to their participation and interview process. The participants were informed that they could leave the study at any time. They were also assured that every measure would be taken to maintain Privacy and confidentiality throughout the research including the use of pseudonyms to protect their identity.

### 2.5 Trustworthiness

The trustworthiness of this study is based on Guba’s criteria (Lincoln & Guba, 1985). Credibility was established through prolonged engagement with participants and extended immersion. Member checking and peer reviewing were done to verify the findings. Triangulation of data collection methods was done using field notes and diaries to collect data. Transferability was facilitated by purposive sampling of HIV-positive women who had experienced domestic violence. Detailed description of the findings and reviewing the literature when interpreting the data was done to support the study findings. An audit trail was conducted to assure confirmability.

## 3. Results

Four main themes emerged from the data including, “regretful past”, “disappointing future”, “loneliness”, and “no other option”, which refer to the condition that the participants experienced in their daily lives due to challenges that mainly stem from the experience of HIV-related domestic violence.

### 3.1 Regretful Past

“Regretful past” was the first sub-them that was extracted from the participants stories. The participants of this study described feelings of yearning and regret over the decisions they made in the past. Participants believed the current hard situation is because they missed many opportunities for having a good life in the past. They also believed that thinking about the past makes them sad and has negative effects on their lives. The following quotation was expressed by one of the participants of this study: “*My current life is a result of my bad choices in the past. My choices were incorrect. I blame myself for this situation*” (participant 2). The participants thought they had not made logical and reasonable decisions in the past. Almost all of the participants’ stories revealed that wrong decision played a significant role in their being infected with HIV. The following manuscript is a typical example of being infected because of inappropriate decision making in the past. *“If I had decided more wisely, I wouldn’t be sick*. *I’m so sorry now and blame myself all the time for this bad fate*” (participant 5). Another participant added: “*I expected the decisions which I made to provide a good fate for me, but I’m a sick person now. It’s too late*” (participant 8).

Being infected with HIV caused the participants’ married life to be far from the life that they had expected and it was challenging. As one of the participants said “*Although my parents were not happy with my decision to marry my husband, I thought I would have a happy life after marriage. But it’s not what I wished. Marriage brought me sickness and a challenging situation”* (participant 3). Participants believed HIV made everything in their lives challenging. The following excerpt is taken from one of the participants. “*Since I became infected, I can’t manage issues easily. I get angry over every little thing and argue with my family. Sometimes our arguments end in physical altercations. Then I think, if I hadn’t made a wrong decision I would never ever have such a bloody situation*” (participant 10). In the story of the participants who have infected husbands, this situation was worse. As another participant expressed “*We* [both participants and her husband] *always have arguments because both of us are sick and can’t understand and tolerate each other”* (participant 4). For participants, their regretful past not only impacted their current lives, but it also influenced the participants’ future. Therefore, they were anticipating a disappointing future. “*I know, I won’t have a good future because my decisions in the past were not made in a correct way”* (participant 9).

### 3.2 Disappointing Future

“Disappointing future” was another sub-theme extracted from the participants’ interviews. In fact, most participants believed their future to be disappointing and they had no motivation to think about it. As an example, one of the participants stated “*I think of death all the time. I think I’m going to die this year or next year. At the beginning of each new year, I ask myself whether I will see the next New Year*” (participant 7). For most of the participants in this study, in addition to regretful past, disappointing future was also one of the major factors which influenced their current life. The following quotations have been taken from the participants of the study. “*I have nothing anymore. My life is nothingness and futile. There is nothing in this life*”. “*I have nothing to lose anymore. My life is not pleasant. I do not like this life*”. “*I’m disappointed about my future. I’ve lost absolutely everything*” (participant 8).

Because of disappointing future and its impact on the current life of the participants, they believed there was no hope for a good future in the current situation. Being hopeless about the future leads the participants to be at risk for domestic violence. For instance, this excerpt is taken from one of the participants: “*when I think I have no hope for the future, I get angry. That’s why I argue with my husband all the time* *and our arguments usually end with fighting*” (participant 2). Some of the participants believed they manage this challenging situation through limiting contact with their husbands. “*I try to stay in my own room to keep myself far from my husband, because we fight when we are together*” (participant 10).

### 3.3 Loneliness

“Loneliness” was another underlying extracted sub-theme from the experiences of all participants. For some of the participants, loneliness was understood as a type of domestic violence. In fact, living in HIV context is associated with such a sense of loneliness that is described by these women as violence. “*Although I live with him, I feel myself alone. For me, this situation* [loneliness] *is a kind of violence*” (participant 6). Some participants were faced with their husband’s infidelity and were left alone by their husband. “*He stays out all night and doesn´t return home. I tell him I’m not feeling well, I need you now. He shouldn’t leave me alone under this condition… I think he does not consider my rights as a wife*” (participant 4). The absence of intimacy and sexual relationships between couples was described by some participants as a feeling of loneliness. These women stated their husbands avoided having sex with them and ignored them. One of the participants described her experience as follows: “*I adorn myself. He doesn’t look at me… We have not had sex for a long time. We’re just spending our days together… I don’t feel that I have a husband. I’m alone all the time… Isn’t this abuse?* “ (participant 8).

The participants who blamed their husbands for infecting them with HIV felt anger and emotional numbing toward their husbands, which led to an increased sense of loneliness in these women. In this regard, one of the participants stated: “*I don’t feel him* [her husband] *anymore. I don’t have any feelings for him. I don’t feel that he is my husband. I loved him before I knew he was sick. I no longer have those previous feelings for him. He infected me with HIV*” (participant 3). Some participants were suffering from the way their husbands behave, leading to greater feeling of loneliness. The following excerpt was expressed by one of the participants. “*It seemed that I didn’t exist for him. I told him I’m not feeling well, he said it’s not my problem… however, I need more of his attention in this situation”* (participant 1).

The experience of most participants in this study indicated that lack of family support resulted in an increased sense of loneliness in the women, which was a major factor leading to argument and conflict between couples. In this regard, one of the participants stated “*my family just rebukes me for my husband’s bad manners. They never help me… They don’t ask me about my needs in my life. I have to struggle with life’s challenges alone*” (participant 2). Another participant added “*Since we have been sick, we need more help but none of my family members help us… when my husband was hospitalized, my husband’s mother didn’t even give us 1000 Tomans for help, even though she works… these problems cause us to argue all the time*” (participant 10). Although the participants were struggling with loneliness in their lives, they insisted on continuing their married life because they had no other choice.

### 3.4 No Other Option

This sub-theme represents the sense of being coerced as a dimension of the experience of domestic violence in HIV context. This experience includes a sense of being coerced by husband and whatever these women have no power to change, including sexual, social and economic coercion. All participants of this study had to continue to live with their violent husbands under challenging conditions because of being HIV-infected. As one of the participants said “*I’m not healthy anymore. I’m a sick person, that’s why I couldn‘t get a divorce from my violent husband, then marry another man … we’re both sick, so we have to live together*” (participant 9). The women infected by husbands did not object to their own husband because they would like to keep the relationship safe. For example, one of the participants stated: “*Although he infected me with HIV, I can’t say anything. My situation is even worse if I blame him… I have to accept it*” (participant 3). However, the participants who were living with uninfected husbands described their lives as painful and they preferred to give up their lives rather than to continue to live. Therefore, attempts to commit suicide were common in these women. As an example, one of the participants tearfully said: “*I swear to God, I’ve given up my life because my life is so excruciatingly painful… I’ve attempted suicide to end my life on many occasions… every day of my life is like death*” (participant 9).

Having a child was one of the main reasons that forced participants to remain with abusive husbands. In this regard, some of the participants said: “*because of my baby, I can’t leave my abusive husband. I don’t want my child to be a divorce baby*” (participant 7). “*I know he* [her husband] *can’t take care of my children. That’s why I must continue to live with him*” (participant 5). Another one added “*My life is going to be harder and harder. You know, I don’t really know which way I have to go. On the one hand, I can’t leave my husband because of my children and my health status. On the other hand, life is painful for me when my husband beats and swears at me*” (participant 8).

To avoid conflict and violence by their husbands, some participants stated that they were forced to have sex with their husbands, despite their lack of interest. In fact, for some participants, having sex with their husband was applied as a way to prevent domestic violence. The following was taken from one of the participants. “*Normally, our arguments and conflict arise if I reject his request for sex. I should accept his request even if I don’t want to*”. Another one says “*I have to have sex with him even in the worst conditions*” (participant 2).

For most of the participants, lack of family and social support was the major obligation to keep their marriage and to accept their current situation regardless of it being a painful life for them. The participants believed that they had no way to escape from that situation. Some participants stated the following: “*I ask myself, I have no family, so where can I go if I get divorced? I have to tolerate my current bloody life*” (participant 10). “*I don’t want to go back to my father’s home. If I go back there, I have to listen to their* [family] *taunts every day. There’s nowhere to go if I want to leave my husband*” (participant 7). The participants who have uninfected husbands were being abused financially because of their HIV-positive status. They had to work to meet their family’s needs without having the right to spend their own money. For instance, one of the participants tearfully said “*I go to work from morning to evening. I work very hard and I get very tired. He just stays home. He does nothing to help me… Nevertheless, he doesn’t allow me to spend my own money. I’m not even allowed to buy a dress for myself … It’s really painful and I can’t tolerate it. I don’t know what to do. If I lived in prison, it would be much better than this life*” (participant 9).

## 4. Discussion

The present study aimed to explore the lived experience of HIV-infected women of domestic violence in Iran. The study results revealed that all participants experienced HIV-related domestic violence. According to UN Women’s report, the experience of domestic violence in HIV-infected women is nearly three times as likely as uninfected women (UN Women, 2012). For HIV-infected women, exposure to multiple traumas, including domestic violence is associated with AIDS-related morbidity (Schafer et al., 2012).

In this study, participants blamed themselves for the incorrect decisions that they made in the past which were mainly about whom they chose as a spouse. They believed that HIV-related domestic violence was the result of their poor choices in the past. They insisted that they were regretful about their past. Having regretful feelings about inappropriate decisions and behaviors in the past can be the basis of abuse and domestic violence. Hsieh et al. (2009) reported most abused women regretted their hasty decision to get married, because they might not have married their husbands if they had spent more time getting to know them deeper (Hsieh, Feng, & Shu, 2009). Some HIV-infected people had regret for past actions that resulted in HIV infection (Cook, McElwain, & Bradley-Springer, 2010). Deep regret for past inappropriate decisions made the participants anxious and it was the starting point for conflict between couples and HIV-related domestic violence.

Disappointing future in the participants was another reason for conflict between couples, leading to HIV-related domestic violence. Despair over the future was mainly described by the participants. The participants were concerned about their future and experienced a feeling of despair over their future due to being HIV-infected. Willis et al. (2014) state HIV makes people worried and desperate for their future (Willis et al., 2014). Being infected with HIV in the participants was compounded by domestic violence and led to lack of hope for the future. People are often motivated by future ambitions to participate in positive behaviors (Mabachi, Dariotis, Goggin, Anderson, & Finocchario-Kessler, 2014) and domestic violence demolishes the hopes of a fortunate future in abused women (Hsieh et al., 2009). Therefore disappointing future was described as a major challenge and it was commonly linked to a sense of loneliness.

Loneliness was considered as a type of HIV-related domestic violence for the participants. Loneliness does not always have the same meaning as being alone. People may live together, but feel loneliness. Loneliness is experienced when meaningful social relationships and emotional bonds are lost (Heravi-Karimooi, Rejeh, Foroughan, & Vaismoradi, 2012). The participants understood loneliness as domestic violence in different ways. For some of the participants, loneliness was a sense of being neglected and lack of husband’s support, so that they felt there was no one in their lives to share their feelings. In some participants, loneliness was a feeling of dissatisfaction with their couple relationship and lack of sexual relationship. In addition, lack of family and social support was considered as an important factor contributing to the feeling of loneliness. The failure to obtain social support from a husband is a significant detriment for HIV-infected women (Edwards, Irving, & Hawkins, 2011). The feeling of loneliness and being neglected exposed participants to ongoing episodes of HIV-related domestic violence. They suffered from mental health problems such as depression, which led to attempts to commit suicide. Depression and suicidal ideation seem to be a common reaction to domestic violence (Hatcher et al., 2013; Loke, Wan, & Hayter, 2012) in HIV-infected people (Kylma, 2005).

HIV-related domestic violence arose from the coercion which participants experienced in relation to sexual activity, social isolation and economic dependency, causing these women to stay in their marriage. Sexual coercion was a common experience in the majority of participants in this study. They were forced to have sex with their husbands to prevent conflict and violence, despite their unwillingness. Some women believed that they had no right to refuse sexual relations with their husbands. This is consistent with reports that some abused women had agreed to sexual intercourse to escape domestic violence (Flinck et al., 2005). The participants believed they were financially dependent on their married life, even though they would prefer to leave their marriage. Fear of rejection and abandonment because of being HIV-infected forces the women to continue living with violent husbands under any condition. Gruskin et al. (2002) state, the abused women avoid leaving their abusive husband if they are not financially independent (Gruskin et al., 2002). However, in the participants who had uninfected husband, their HIV-positive status allowed their husbands to take considerable advantage of them. This financial abuse was a very painful situation for the participants. However, they could not do anything to escape from this situation because their husbands threatened to disclose their HIV status to others. In Iran, it is not surprising that women endure domestic violence in silence, because the Iranian family structure is still dominated by patriarchal values (Abadi et al., 2012). This situation can be more complicated for women who are HIV-infected. In addition, given that one of the most common activities resulting in HIV infection is risky sexual behaviors, and sexual activity outside of marriage is considered to be a criminal act in Islamic law (Mohammadpour, Parsa Yekta, Nikbakht Nasrabadi, & Mohraz, 2009), the stigma against HIV-infected people that is more intense in Muslim countries including, Iran, (Rahmati-Najarkolaei et al., 2010) indirectly forces the HIV-infected women to endure husband’s violent behaviors and all forms of abuse.

According to the civil laws in Iran, women can own their properties (Attaie Ashtiani, 2008). Therefore, if women earn money, it is their own property and they can keep it for themselves. However, the participants were financially abused due to being HIV-infected. They get abused to earn money to support the family. Moreover, having a child caused the participants to be unable to leave their husbands. The abused women have to stay with the family for the children in order to provide them with a family (Loke et al., 2012). When abused women make the decision to leave their husbands, they often consider their children prior to considering their own needs (Hsieh et al., 2009). In Iran, custody of children is mainly granted to the father after divorce (Pourasadi & Hashemi, 2012) which forces the women to remain trapped in a violent relationship, tolerating domestic violence.

This study focused on a limited number of HIV-infected women in Iran’s cultural context. Therefore, the findings of the current study cannot be generalized to other societies and cultures. However, results of qualitative research are not intended to be generalized. In addition, due to the stigma with HIV and domestic violence in Iran, Women’s reports may have been influenced by social desirability bias.

## 5. Conclusions

This study provided an understanding regarding what abused HIV-infected women experience during their lives. The findings of the study suggest that Iranian women experience HIV-related domestic violence, and HIV infection is an influencing risk factor for domestic violence. The findings have implications for health care providers to raise awareness and understanding with regard to HIV-infected women’s experiences of domestic violence to identify and support these women. Understanding the experiences of abused HIV-infected women is necessary to develop appropriate, culturally sensitive interventions to prevent domestic violence and to meet the needs of HIV-infected women in Iran. In addition, it is recommended that legal and policy measures be provided to support women with HIV infection, experiencing domestic violence.
